# Uncertainties of forest area estimates caused by the minimum crown cover criterion —a scale issue relevant to forest cover monitoring

**DOI:** 10.1007/s10661-012-2950-0

**Published:** 2012-11-11

**Authors:** Paul Magdon, Christoph Kleinn

**Affiliations:** Chair of Forest Inventory and Remote Sensing, Burckhardt-Institute, Georg-August-Universität Göttingen, Büsgenweg 5, Göttingen, 37077 Germany

**Keywords:** Forest definition, REDD, Deforestation, MRV, Harmonization, Forest edge

## Abstract

Defining “forest land” is a complex issue and has been discussed for decades. Today, a confusing multitude of definitions of forest land are in use making comparison of forest area figures difficult. But currently, comparability is receiving much attention when it comes to install market mechanisms for ecosystem services. Minimum crown cover is among the most frequently employed criteria of forest definitions. However, the size of the reference area on which the crown cover percent is to be measured is usually not defined. But how does a change of the size of the reference area affect the derived forest cover? In this study, we analyze the interactions of the crown cover threshold and the size of the reference area. We start with analyzing the interactions using a simple geometric model of the forest edge. Then, we extend the analysis by simulating artificial landscapes where we study how the interaction is affected by different degrees of forest fragmentation, crown cover proportion, and spatial resolution of the data source used. The simulation showed that large differences in forest cover (>50 %) may result for a fixed crown cover threshold value, just by changing the size of the reference area, where the magnitude of this effect is a function of the chosen threshold value and the spatial configuration of the crowns. As a consequence of the findings, we see an urgent need to complete forest definitions by defining a reference area in order to reduce uncertainties of forest cover estimates.

## Introduction

### Background

Forests are receiving much attention in various functions; among them are their role as carbon sinks and sources, as home to the greatest terrestrial biodiversity, and as a basis for the livelihood of many rural dwellers. Forests are in the core of international policy processes where reliable data and information become a crucial issue for policy formulation and decision making. For example, the United Nations Framework Convention on Climate Change (UN-FCCC) requires signatory nations to trace and report their greenhouse gas emissions which include the forestry sector where monitoring of the area of forest lands and its changes is a major concern. Currently, the Conference of the Parties to UN-FCCC is requesting the Subsidiary Body for Scientific and Technological Advice to develop necessary modalities for measuring, reporting, and verifying (MRV) anthropogenic forest-related emissions including forest area changes (UNFCCC 2010). Changes in the area of forest land can only be determined and monitored when robust, transparent, replicable, and long-term national forest monitoring systems are in place (Holmgren et al. 2008). Here, the seemingly simple question of the definition of forest land becomes crucial: how can “forest land” be defined in an operational and meaningful manner, such that forest and non-forest land can efficiently be distinguished and that the corresponding areas can be delineated unambiguously (see Colson et al. 2009; Kleinn et al. 2002; Mathys et al. 2006)? For clarity, throughout this paper, we make the distinction between “forest” (being a set of trees in a certain number, density, and spatial arrangement) and “forest land” (the land where the set of trees that constitute a forest is located). To simplify matters, we use the term “forest cover” equivalent to “forest land cover.”

For many decades, forest politicians, forest ecologists, forest monitoring experts, silviculture experts, and probably other experts have come up with a multitude of forest definitions for different purposes: legal definitions for issues such as land allocation and tax assessment, ecological definitions for habitat studies, silvicultural definitions for management and stand treatment purposes, as well as definitions for inventory and mapping for both ecological and production management purposes. The most comprehensive list of definitions of forest land of all kinds is probably that by Lund (2011).

In the current discussion on instruments to monitor changes in forest lands and to foster carbon sequestration on forest lands, the issue of defining forest has gained new momentum. According to the IPCC guidelines (Penman et al. 2003), forest carbon balances are derived from two major inputs: (1) emission factors (e.g., carbon per unit area) and (2) activity data (e.g., forest area changes). As a consequence, the assessment of the area of forest land is considered an essential prerequisite for quantifying forest carbon dynamics, where a meaningful further subdivision into different forest types would be a next step. This is especially relevant when it comes to payment schemes and to the establishment of market mechanisms as the definition of forest land will determine the countervalue of the traded certificates.

### Elements of forest definitions

Criteria used in forest definitions can be broken down to quantitative and qualitative ones (Kleinn 1991, 1992, 2001; Lund 2002; Vidal et al. 2008) which refer to both characteristics of forest and of forest land. Usually, a comprehensive forest definition includes threshold values for the following quantitative variables: (1) minimum area, (2) minimum crown cover percent, (3) minimum tree height, and (4) minimum width. Qualitative criteria specify, for example, how to deal with special features such as roads, creeks, and clear-cuts within otherwise tree-covered areas; whether plantation forests and/or plantations of “non-forest” trees (e.g., fruit trees, rubber trees) are to be included; whether palm and bamboo vegetation should count as forest land; and how to deal with tree cover in the presence of other land uses.

Here, we focus on the criterion crown cover percent, which is the proportion of the land covered by the vertical projection of tree crowns, overlaps not counted (Geschwantner et al. 2009; Jennings et al. 1999). It is one of the most commonly used criteria in national and international forest definitions with threshold values ranging from 10 to 100 % (see Lund 2011). To observe the variable crown cover percent, two data sources are commonly employed, either individually or combined: (1) field observations and (2) remotely sensed observations. These two data sources are briefly discussed below.

### Two major data sources to quantify crown cover: (1) field observations and (2) remotely sensed observations

Sample-based field inventories are a common approach to directly observe tree and forest variables. Wherever possible, the forest/non-forest decision is made before going to the field in order to save resources. However, if such a decision cannot be made a priori, the criteria of the forest definition need to be applied in the field; this includes the observation of crown cover at the sample location. Determining crown cover percent in the field, however, is difficult. There are numerous, time-consuming field-based techniques including line transect sampling (Gregoire and Valentine 2008; Ko et al. 2009), sighting tubes (Korhonen et al. 2006), spherical densitometers (Lemmon 1956), and direct measurement of tree crown dimensions. Terrestrial remote sensing is also applied, either from hemispherical photographs (Korhonen and Heikkinen 2009) or from terrestrial laser scans (Korhonen et al. 2010). However, given the geometrical properties (the central perspective) of these techniques, canopy closure is observed rather than canopy cover as pointed out by Jennings et al. (1999).

From optical remote sensing, only a limited number of variables can directly be observed, namely, the wavelength and intensity of reflected electromagnetic radiation integrated over a defined area on the ground (the so-called IFOV = the ground-projected “instantaneous field of view” = the ground area covered by one pixel) for a defined number of spectral ranges, and the relative position of that pixel. All other variables, including crown cover, are not directly observed but modeled on the basis of these observations, their variability, and spatial characteristics. These characteristics depend on the spatial resolution of the sensor used, among other criteria. Strahler et al. (1986) distinguished two different qualities of spatial resolutions when observing specific objects of interest (e.g., tree crowns): low resolution (L-resolution) where the pixel size is larger than the objects of interest and high resolution (H-resolution) where pixel size is smaller. Many remote sensing systems used for large-area forest cover monitoring on a wall-to-wall basis are in the L-resolution domain, including the 1-km resolution data of Advanced Very High Resolution Radiometer (DeFries et al. 2000), the 250- and 500-m resolution data of Moderate Resolution Imaging Spectroradiometer (Hansen et al. 2002, 2003), the 300-m resolution data of Envisat’s Medium Resolution Imaging Spectrometer (Berberoglu et al. 2009), or the 30-m resolution data of Landsat (McRoberts 2006). Individual tree crowns cannot be identified in these images nor can the ratio between ground and crown pixels be directly observed. Here, the crown cover percent for a pixel is inferred from the intensity of vegetation response over the pixel area. The potential of analyzing individual tree crowns for large areas arises from a new generation of H-resolution satellite sensors with pixel sizes in the meter (e.g., RapidEye, Ikonos, SPOT 5, TerraSAR-X, Radarsat) and submeter (e.g., Worldview I&II, Quickbird, Ikonos) range. Using H-resolution sensors, individual tree crowns can be identified and crown cover measured; this is contrary to the application of L-resolution sensors. Therefore, when changing from L- to H-resolution imagery for forest mapping, not only the spatial resolution is relevant but also a second scale component needs to be considered: the reference area which will be elaborated in the following section.

### The reference area—a critical scale issue for measuring crown cover percent

A dimensionless sample point is either covered by a tree crown or not. To determine a ratio (crown cover percent), it is necessary to define a reference area around that point. The area around a point on which a value of a variable is determined is known from geo-statistics as the support area (Matheron 1984) and such a variable is called a regionalized variable. It is well established that the size of the support area determines the distribution statistics of that variable (Myers 1994). The crown cover is such a regionalized variable and its support area is the reference area on which the crown cover is measured. But how is the reference area defined in a forest inventory?

The size and shape of the reference area are implicitly determined when measuring crown cover percent: if it is measured in the field using a dot grid, the plot size on which the dot grid is laid out determines the reference area (see Korhonen et al. 2006). If terrestrial remote sensing is used, e.g., hemispheric photographs, view angle of the devices and stand height define the reference area (see Korhonen and Heikkinen 2009). In L-resolution remote sensing imagery, the pixel size is larger than a tree crown. Thus, the spectral response represents a mixture of tree crowns and other land cover classes and therefore crown cover cannot be measured. A common approach to estimate crown cover in L-resolution data is to build a model which predicts crown cover percentage for each pixel on the basis of the spectral signature (e.g., Hansen et al. 2002). Using such a model approach, the size of the pixel itself defines the reference area for crown cover estimation. In H-resolution imagery, the pixel size is smaller than the tree crowns. Thus, a pixel is either covered by tree crowns or not which results in a binary classification. For simplification, we neglect here the confounding effect from mixed pixels at the edge of tree crowns. Therefore, a reference area larger than one pixel, usually a square window of pixels, needs to be defined. That means that, in H-resolution data, the reference area is different from the spatial resolution and introduces a second scale component which needs to be considered and defined when observing crown cover percent.

The effect of spatial resolution on image interpretation in the L-resolution domain has been a subject of numerous studies in the context of land cover mapping (Atkinson and Curran 1995; Nelson et al. 2009; Zheng et al. 2009; Woodcock and Strahler 1987). Mareceau and Geoffrey (1999) give a detailed literature overview of the effect of changing the support to which they refer to as “modifiable area unit problem” MAUP. Nevertheless, an analysis of the effect of the reference area size in H-resolution data when determining a crown cover percent appears not to have been the subject of specific research. The only studies in the forestry context so far appear to be those of Kleinn 1991; 2001 who observed on simple examples how a variation in the reference area size affects the resulting forest cover estimate. He found an interesting and potentially substantial impact of the reference area that interacted with the threshold value for minimum crown cover and with forest spatial pattern.

### Objectives

Over the past decade, there have been intensive discussions concerning the compatibility of forest definitions (Lawrence et al. 2010; McRoberts et al. 2010; Vidal et al. 2008). While much attention is given to the definition of threshold values for the quantitative criteria in forest definitions (Neeff et al. 2006; Nelson et al. 2009; Sasaki and Putz 2009; Verchot et al. 2007; Zomer et al. 2008), there is hardly any description nor definition of the corresponding measurement rules, which usually is an integral part of a definition when used in an inventory protocol.

In the present study, we analyze such a measurement rule for the commonly used criterion “minimum crown cover” which is the size of the reference area. We perform an in-depth analysis of the relations between minimum crown cover and the size of the reference area. We are interested in the measurement process of these variables from a remote sensing perspective with the following specific research questions:

1. How do the variables “crown cover percent” and “size of the reference area” interact when forest cover is estimated in H-resolution images?
2. Is this interaction influenced by (a) the composition of the landscape, (b) the fragmentation status of the landscape, and (c) the spatial resolution at which the landscape is observed?
3. Can the interaction effects from crown cover percent and size of the reference area be modeled in order to predict changes in the estimated forest cover if the values of one or both criteria are changed?

We structured the text as follows: First, we illustrate the interaction of the size of the reference area with a simple geometrical model. Then, we use artificially generated tree crown cover maps with different total amount of crown cover, fragmentation patterns, and spatial resolutions to study potential impacts on the interaction of crown cover threshold and size of the reference area. In a last step, we build linear models to predict differences in forest cover as a function of the reference area size and crown cover threshold.

## Methods

### Generating neutral landscapes for simulation

To study the effects of changes in reference area size on forest land figures in complex landscapes, we use artificial maps with only two classes: crown and non-crown. Three factors are systematically varied which constitute major map properties: (1) the proportion of crown pixels (*p*), (2) the spatial pattern of crown pixels (*α*), and (3) the spatial resolution.

**Fig. 1:**
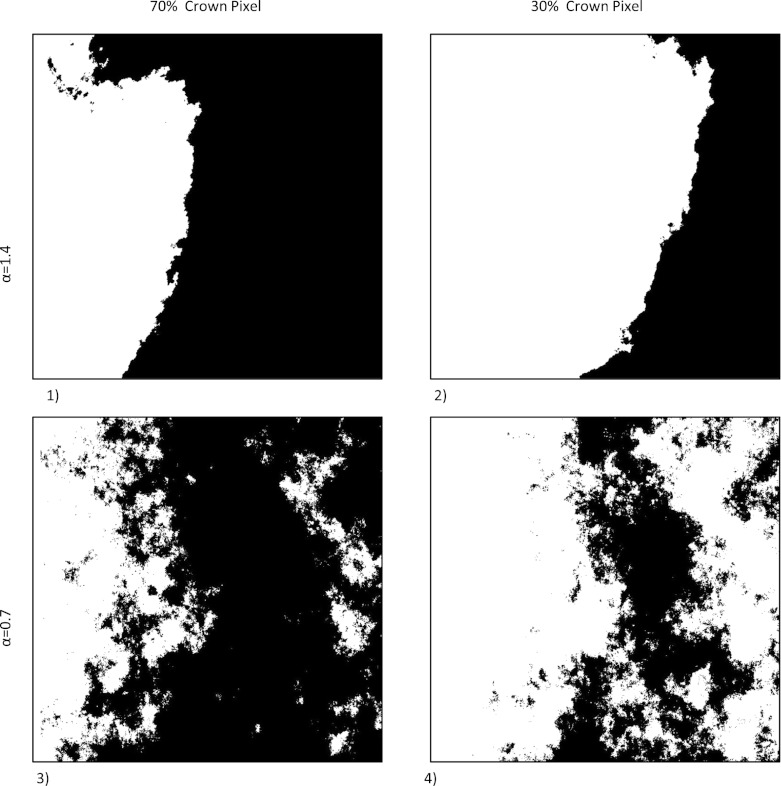
Examples of the four binary landscape types used in the simulations (*black* = crown pixel, *white* = non-crown pixel) with two levels of overall crown cover (*p*) and two types of spatial pattern (characterized by *α*): *1*, compact with large proportion of crown pixels [*α* = 1.4, *p* = 0.7]; *2*, compact with small proportion of crown pixels [*α* = 1.4, *p* = 0.3]; *3*, highly fragmented with large proportion of crown pixels [*α* = 0.7, *p* = 0.7]; and *4*, highly fragmented with small proportion of crown pixel [*α* = 0.7, *p* = 0.3]

Gaussian random fields are a convenient starting point for spatial models of binary data as spatial dependencies can easily be modified through the covariance model, and simulation algorithms are available in the statistical software package R (Schlather 2001). To vary the spatial distribution of the tree crowns, we generated landscapes with distinctly parameterized covariance models. For all landscapes, we used a one-parametric form of the generalized Cauchy model as the covariance model (Eq. 1). We selected the Cauchy model, as it leads to realistic-looking crown cover maps (see Fig. 1) and allows to control the degree of fragmentation using one parameter only.

1 $$ C(h)=(1+|h|^\alpha)^{-\frac{1}{\alpha}} \label{eq:1} $$

This model describes the covariance *C*(*h*) of two observations at distance *h*, where *α* defines the fractal dimension *D* according to the Hausdorff dimension (Gneiting and Schlather 2004). The fractal dimension of the graph of the corresponding Gaussian random field equals 3 − *α*/2 (Gneiting and Schlather 2004). It is a measure of patch complexity and an indicator for the fragmentation status of the landscape. Using low *α*-value results in more fragmented and scattered landscapes, high *α* values produce more compact shapes with larger patch sizes and smoother edges. In this study, we include “highly fragmented landscapes” with an *α* value of 0.7 and “compact landscapes” with an *α* value of 1.4.

For simulating different total numbers of crown pixels *p* in the landscape, the continuous Gaussian random fields were transformed into binary images (crown/non-crown), using the 0.3 and 0.7 quantile of the normal distribution, resulting in landscapes with 30 and 70 % crown pixels, respectively. In total, a number of *n* = 100 different maps (also called “repetitions” in what follows) were generated for each of four basic landscape types (see Fig. 1).

To analyze the effects of different spatial resolutions, we artificially degraded the resolution in three steps by aggregating the crown maps using a majority rule within a 3×3 window. We started with maps of 2,700×2,700 pixels (high resolution) and degraded them to 900×900 pixels (medium resolution) and 300×300 pixels (low resolution). However, we are aware that changing the spatial resolution of a true sensor would also result in a different spectral pattern. A simple aggregation as in the simulation will, therefore, not fully mimic the effects of changes in the spatial resolution of sensors and the corresponding results need to be interpreted with care.

### Workflow for the forest/non-forest distinction

**Fig. 2:**
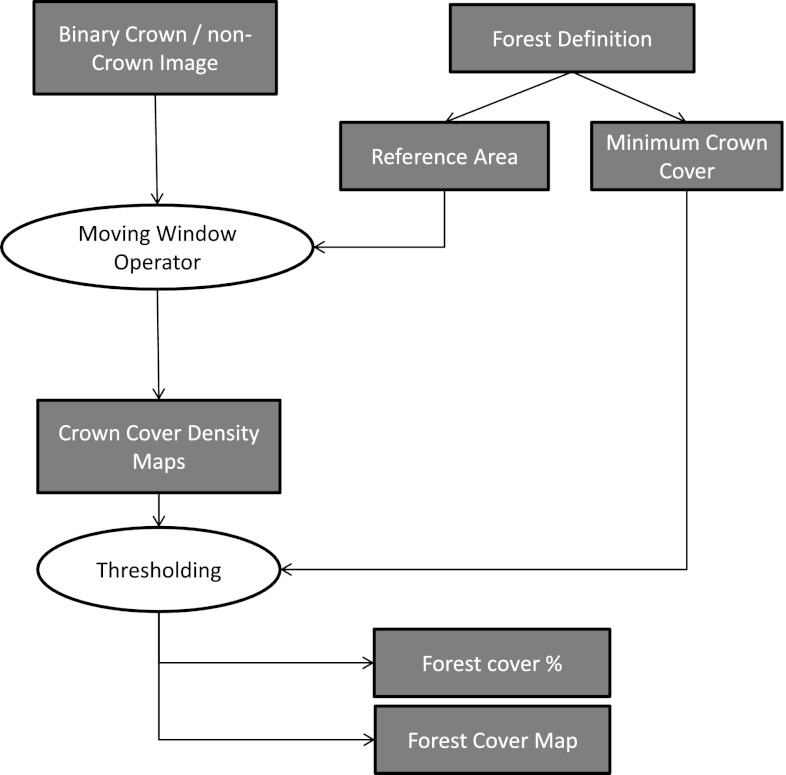
Workflow for classifying forest/non-forest based on the minimum crown cover criterion when analyzing binary crown cover maps

Figure 2 illustrates the image processing workflow for generating forest cover figures from simulated binary crown maps applying the minimum crown cover criterion. We started with the binary crown/ non-crown image assuming that the maps are an error-free classification into tree crown and non-tree crown pixels. We are aware that, in practice, image classification issues make that procedure more difficult, and more criteria would need to be considered than only minimum crown cover. However, for this study, we aim to analyze the impact of minimum crown cover on forest cover estimates in an isolated manner.

From the binary crown maps, a decision has to be made for every pixel whether it is forest or non-forest, which is done in two steps. In the first step, the crown cover percent is determined in a reference area of a defined number of pixels around the pixel in question. The crown cover percent is the ratio of crown pixels to total number of pixels in that area. This proportion is assigned to the pixel in question, and the same procedure is applied to all pixels using a *moving-window* operator. The result is a map of proportions in a first step, which we refer to as crown cover density (CCD) maps. Pixels with reference areas that are not completely within the image boundaries were excluded from further analysis here. As different map sizes can influence the results, we clipped all density maps using the largest reference area size so that all forest maps had the same extent.

Ten square reference areas with a side length of 3, 7, 11, 15, 19, 23, 27, 31, 35, and 39 pixels were used. This size refers to the low-resolution maps (300×300 pixels) and was then adapted to the higher resolution levels so that it represented the same original extent in each crown map: for example, the reference area size of 3×3 = 9 pixels in the low resolution was increased to 9×9 = 81 pixels for medium resolution and to 27×27 = 729 pixels for high-resolution maps.

The second step was assigning a threshold to the CCD maps, so that the minimum crown cover criterion can easily be applied to decide whether a pixel qualifies as forest or not. The forest edge then follows the line where crown cover percent falls below the threshold given by the forest definition. To analyze interactions between the threshold value and the size of the reference area, we produced binary forest maps using ten different minimum crown cover thresholds *t* (0.1, 0.2,..., 1).

In the end, the simulations included *n* = 100 random repetitions of binary crown maps for each of the four landscape types that were observed at three resolution levels and were classified utilizing 10 sizes of reference areas. This resulted in 12,000 CCD maps (100 repetitions × four landscape types × three resolutions × 10 sizes of the reference area). From these CCD maps, forest maps with 10 different minimum crown cover threshold values were generated resulting in 120,000 forest maps in which the target variable forest cover was observed. Analyzing large raster datasets with moving-windows techniques is computationally extremely demanding as the neighborhood of each pixel in a raster needs to be analyzed separately. The number of *n* = 100 repetitions as used here is a compromise between accuracy and available resources. All simulations and the analysis were done using the open source software R (R Development Core Team 2011) and GRASS (GRASS Development Team 2011).

### Predicting effects based on changes in the reference area size

Previous studies from Kleinn (2001) indicated systematic effects of crown cover threshold values and corresponding reference area sizes on forest cover estimates. In addition to describing these effects, we were interested whether they can be modeled and how a model would perform for different landscape patterns. As a starting point, we randomly selected one of the *n* = 100 maps and fitted a simple linear model with the threshold value *t* as the only predictor (see Eq. 2). We repeated this for each of the 10 sizes of reference areas and for each of the four landscape types (as described in Fig. 1). In total, 40 linear models were fitted by least squares regression.

2 $$ y=\beta_0+\beta_1\times t +\epsilon_i \label{eq:2} $$

The basic geometric simulation (see Section “Basic geometric simulations of the forest edge”) showed that the size of the reference area interacts with the value of *t*. For straight forest edges and *t* = 0.5, no effects of the reference area size are expected, whereas for $t\not=0.5$, the effect of the reference area size is increasing with increasing values of |*t* − 0.5|. Based on this observation, we extended the previous model by including the size of the reference area using a term which reflects the observed interaction. Equation 3 gives such a model where *y*_*i*_, the forest cover in landscape *i*, is a function of the crown cover threshold *t* and the reference area size *r*. *β*_0_, *β*_1_, and *β*_2_ are regression coefficients and *ε*_*i*_ is the residual error.

3 $$ y_i=\beta_0+\beta_1\times t+\beta_2\times r(0.5-t) +\epsilon_i \label{eq:3} $$

To compare the extended model (Eq. 3) to the basic model (Eq. 2), we fitted it to the same landscape and evaluated the quality of both models based on residual plots and the coefficient of determination. In the second step, we analyzed the predictive power of the extended model (Eq. 3). Therefore, we pooled all landscapes of one type and fitted one global model where we evaluated the goodness of fit using the coefficient of determination.

## Results

### Basic geometric simulations of the forest edge

**Fig. 3:**
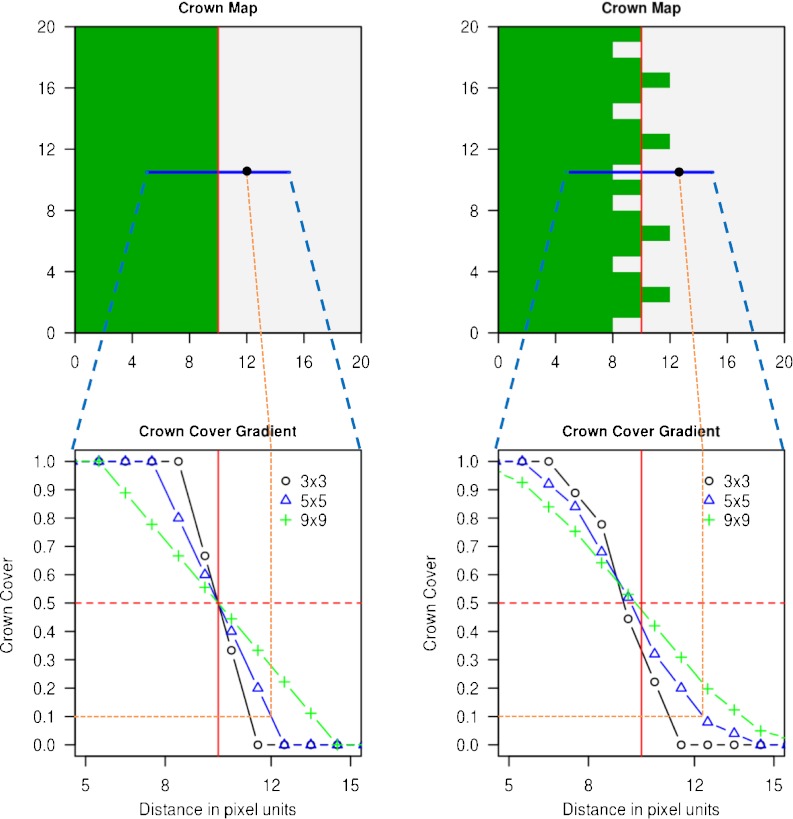
Schematic plot of a straight (*left*) and irregular (*right*) forest edges in a map (*top*) and crown cover gradient at the marked transects for three different square reference areas (*bottom*). The *fine dotted lines* give an example of how to read this graph: for a minimum crown cover of 0.1 and a reference area of 5×5 units, the *vertical fine dotted line* points to the locations in the above maps where the forest edge is located (i.e., the transition from forest to non-forest)

To examine the effects of implementing a forest definition with different sizes of reference areas, we studied two simple spatial configurations of a forest edge as shown in Fig. 3. On the left-hand side, a straight line separates crown covered (dark) from non-crown covered land (light). On the right-hand side, there is an irregular boundary separating crowns from open land. We now applied different reference areas (moving windows) to these situations, moving them along the transect as shown in Fig. 3 from crown covered to the open area. The crown cover percentage in the reference areas gradually change from 100 to 0 %, and this change is different for varying sizes of reference areas and for both shapes of the boundary as depicted by the gradient lines in Fig. 3 (bottom).

For the straight forest edge, all gradient lines intersect at a crown cover threshold *t* = 0.5. With the approach presented here, a pixel qualifies as forest if the surrounding reference area complies with the minimum crown cover criterion; for *t* > 0.5, the forest edge “moves into” the crown covered land, and for *t* < 0.5, it is shifted outside the crown covered land. The displacement of the forest edge is in this case a linear function of the size of the reference area. For an irregular forest edge, as shown in the right-hand side of Fig. 3, the relation between *t* and the reference area follows an inversely S-shaped curve; the lines for the different reference areas do not intersect at *t* = 0.5 and they do not have one single common intersection.

This basic geometric model illustrates the effect of the size of the reference area and points to landscape spatial configuration as an important factor when defining forest by a minimum crown cover threshold.

### Edge effects in artificial crown maps

**Fig. 4:**
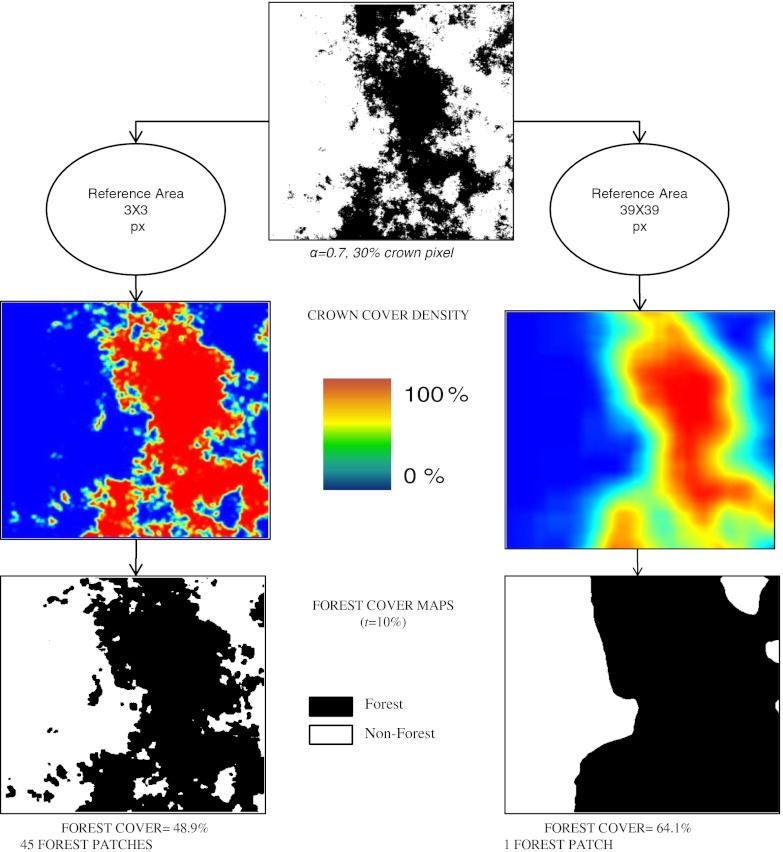
Results of forest cover mapping for a highly fragmented landscape (*α* = 0.7) with 30 % crown pixels and a minimum crown cover threshold of *t* = 0.1

The simulations on artificial crown maps confirmed what has been observed in the simple geometrical model and allows a more detailed insight into the effects of relevant factors. The CCD and forest/non-forest maps shown in Fig. 4 were produced from the same crown map using two different reference area sizes, but identical *t* = 0.1. Figure 4 shows that both forest cover and spatial pattern are affected. As expected, we observed that, for large reference areas, the number of patches was significantly lower as an increasing size of the reference area leads to a generalization of the forest cover maps.

**Fig. 5:**
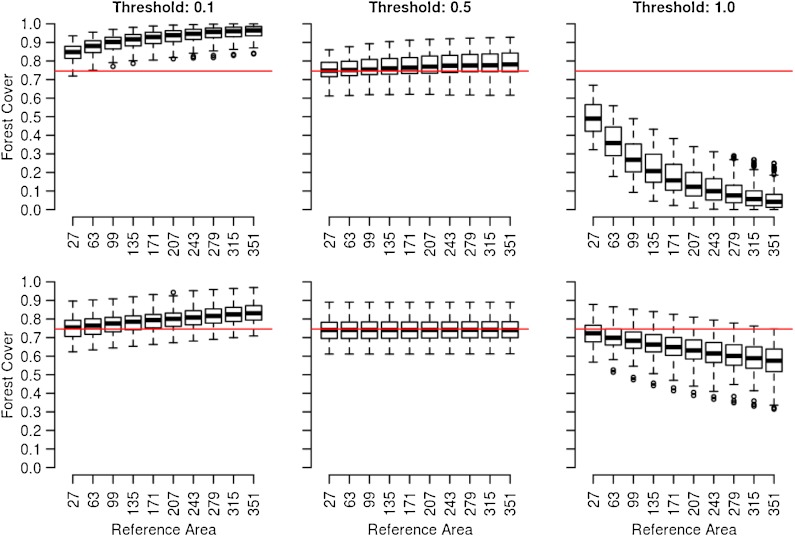
Box–whisker plots of forest cover for different reference areas and crown cover thresholds for a highly fragmented landscape (*α* = 0.7, *top row*) and a compact landscape (*α* = 1.4, *bottom row*). The *horizontal line* marks the average proportion of crown pixels in the crown maps. The *box* marks the upper and lower quartile with whiskers extending to the most extreme data points but no more than 1.5 times the interquartile range

Figure 5 gives the results more explicitly by summarizing the simulation results for *n* = 100 landscapes. For low threshold values (*t* = 0.1), forest cover increases with increasing reference size. For high threshold values (*t* = 1.0), the opposite trend is observed. These trends were also described by Kleinn (1991, 2001) in general terms. For the threshold value of *t* = 0.5, the forest cover approximates the true proportion of crown pixels. It has to be mentioned that due to the clipping of the CCD maps as described in section “Workflow for the forest/non-forest distinction”, there is a difference between the true proportion of crown pixel in the studied crown maps and in the simulated random fields (30 and 70 %). The true proportion of crown pixel in the crown maps is 74.6 % when averaged over all landscapes. For *t* = 0.5, changes in the size of the reference area resulted in only minor changes of forest cover values. Similar trends can be observed for maps with a lower crown cover of 30 % (results not given here).

Comparing fragmented (top row) and compact (bottom row) landscapes in Fig. 5, the following effects of spatial configuration are observed: (1) the effect of the crown cover threshold on forest cover is greater in highly fragmented maps than in compact maps, (2) the effect of the size of the reference area on forest cover is greater in highly fragmented landscapes than in compact ones, and (3) the effect of the size of the reference area follows a curve shape for the fragmented maps and a close to straight line for the compact maps.

### Effect of spatial resolution

**Fig. 6:**
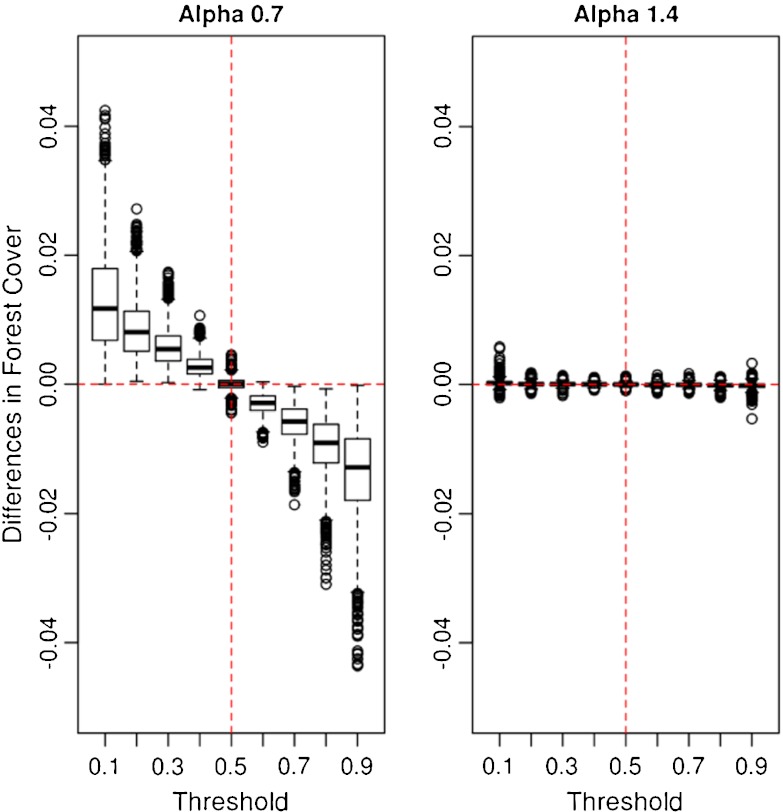
Differences of forest cover resulting from subtracting low-resolution results from high-resolution results. *Left*: For highly fragmented landscapes. *Right*: For compact landscapes. Obs.: Due to the very small overall differences, the *y*-axes have been stretched considerably

To analyze the effects of spatial resolution, we compared the forest cover resulting from low- and high-resolution crown maps. Only minor differences were observed in total forest cover, with a mean difference of 0.7 % for all forest maps. A maximum difference of 26.1 % was found for *t* = 1.0 (not presented in Fig. 6) and a minimum of 0 %. The box–whisker plots in Fig. 6 give the distribution of the differences in forest cover as a function of *t* indicating that the effect of the resolution depends on the threshold value and on the degree of fragmentation. For compact landscapes (Fig. 6, right), no differences are observed. Even though the differences are small for highly fragmented landscapes, a trend is evident (Fig. 6, left). For *t* < 0.5, differences are positive, indicating that more pixels are classified as forest in the high-resolution images than for low-resolution images. For *t* > 0.5, the contrary is observed. Interestingly, *t* = 0.5 again indicates a turning point, here for the spatial resolution, with no significant differences between forest covers estimates.

### Predicting effects based on changes of reference area size

Predicting differences in forest cover as a function of the crown cover threshold value and the reference area size may be an important tool for making forest cover figures comparable and to illustrate the relevance of this topic. Motivated by the findings from the geometric model and the analysis of artificial crown maps, we were interested to analyze whether such a prediction can be done by a simple model. In the first step to build a prediction model, we fitted 40 simple linear models (Eq. 2) using the simulation results of one randomly selected crown map.

**Fig. 7:**
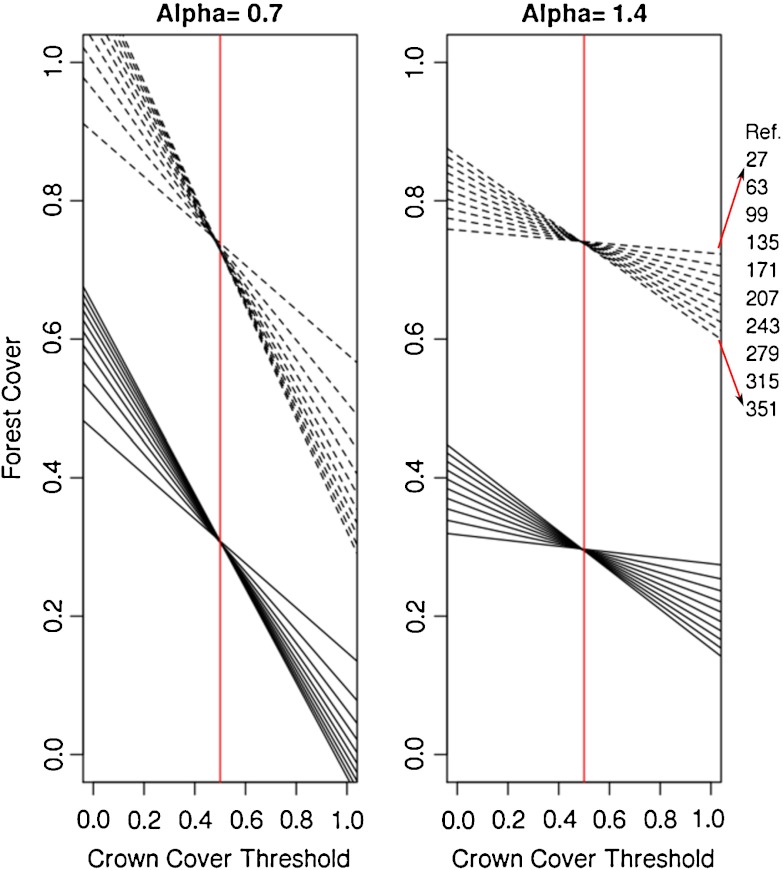
Fitted linear models (Eq. 2) for each of the 10 sizes of reference are plotted for landscapes with 30 % crown cover (*bold lines*) and 70 % crown cover (*dashed lines*) with a highly fragmented spatial pattern (*left*) and a compact pattern (*right*). The order of the regression lines is given at the right margin, where the size of the reference area refers to the side length of a squared reference area given in number of pixels

The regression lines in Fig. 7 show that an increase in *t* leads to a decrease of the forest cover for all studied situations. This effect, indicated by the slope of the regression line, is highest for large reference areas and for highly fragmented crown maps. For all models, both regression coefficients were significant at a *p* level of 5 %.

All regression lines intersect at a *t* value of approximately 0.5 which indicates a small effect of the reference area size for *t* = 0.5 but increasing effects for values departing this intersection point.

**Fig. 8:**
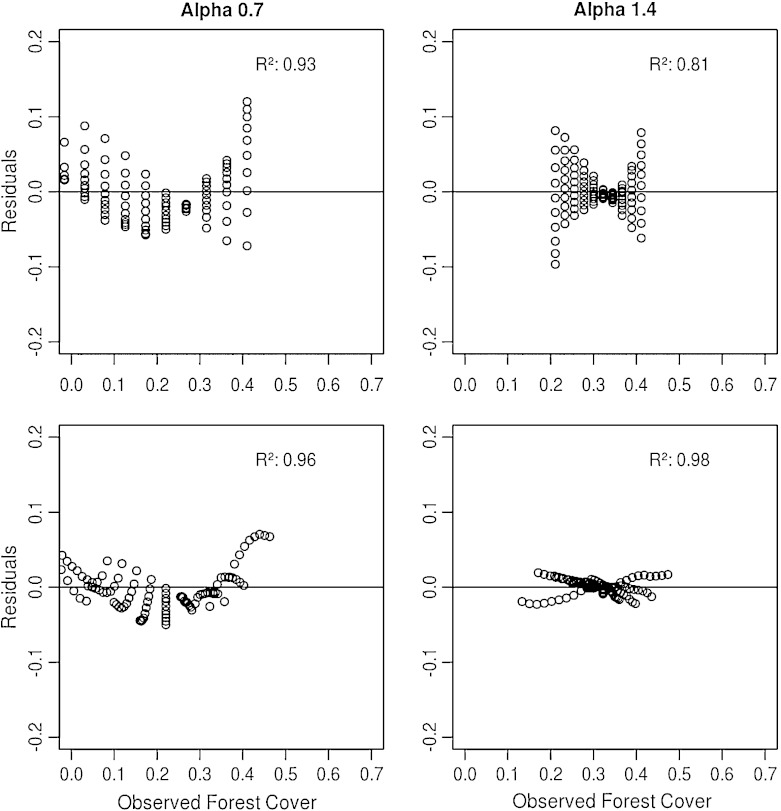
Residual plots of the fitted basic model (Eq. 2) (*upper row*) and the extended model (Eq. 3) (*lower row*) for a compact (*right*) and highly fragmented (*left*) landscape with 30 % crown cover

The effect which was already observed in the simple geometric model is also present in the empirical spatial analysis. In the second step towards formulating a prediction model, we used this observation and extended the basic model (Eq. 2) by a term which incorporates this specific interaction between the reference area and the crown cover threshold (Eq. 3). To evaluate the model quality, we analyzed the residuals as shown in Fig. 8, where the residuals of both models (Eqs. 2 and 3) are compared for compact (right) and highly fragmented (left) landscapes. A clear horizontal pattern of the residuals from the basic model (Eq. 2) can be observed, which reflects the missing term for the size of the reference area. For the residuals from the extended model (Eq. 3), a curved pattern indicates that the linear model used is not appropriate as the residuals are correlated. Although both models show correlated residuals, a better performance is observed for the compact landscapes which show a reasonable fit (*R* ^2^ = 0.98). Again, these differences reflect the findings of the simple geometric model, which indicated that a linear model is only appropriate for straight forest edges. The irregularities of the forest edge are less for compact crown maps, and thus we restricted the further analysis to those maps.

In practice, only one forest map is usually available for a given study area. Fitting the model based on multiple forest maps, as we have done with the simulation, is impossible under those circumstances. We therefore tested if a general model can be applied to various forest maps. One extended model (Eq. 3) was fitted to the pooled results of *n* = 100 compact crown maps (*α* = 1.4) with 30 and 70 % crown pixels; the results are given in Table 1.

**Table 1:** Estimated model parameters of the extended model (Eq. 3) fitted to the pooled dataset of *n* = 100 compact landscapes (*α* = 1.4)

Crown cover	Parameter	Estimate	Standard error	*p* value
30 %	*β*_0_	0.312	0.002591	$<2\textrm{e}^{-16}$
	*β*_1_	−0.0333	0.004881	$<8.94\textrm{e}^{-12}$
	*β*_2_	−0.000763	0.000022	$<2\textrm{e}^{-16}$
70 %	*β*_0_	0.75	0.002429	$<2\textrm{e}^{-16}$
	*β*_1_	−0.0208	0.004576	$<5.61\textrm{e}^{-12}$
	*β*_2_	−0.000683	0.000021	$<2\textrm{e}^{-16}$

The intercepts (*β*_0_) are close to the true proportion of the crown pixel within the landscape. Compared to the high quality of the fit for individual crown maps, the coefficients of determination for the general model are low (*R* ^2^ = 0.39 for 30 %, *R* ^2^ = 0.36 for 70 % crown cover) indicating that a considerable proportion of the variance of the different forest maps cannot be explained by the model.

## Discussion and conclusions

Classification of land cover and/or land use is a standard task in geography and cartography. It essentially serves to depict and report complex spatial patterns and also produce simplified illustration. The concept of land cover classes, however, is inherently vague, and this vagueness tends to persist even when steps are taken to precisely define those land cover classes (Bennett 2001). Defining forest land is such a problem and has been discussed for decades. Here, vagueness does little harm as long as the produced maps are used for illustrative purposes or for a general overview. However, if maps are analyzed quantitatively, such vagueness is difficult to handle, and in cases where forest area is directly linked to high economic values, as it is the case for example in the Reducing Emissions from Deforestation and Forest Degradation (REDD) Program, vagueness is hardly acceptable as an element of a methodological approach.

In this study, we demonstrated and illustrated that the common approach to define forest land, by specifying threshold values only, is incomplete as long as measurement rules are not defined. We study the case of minimum crown cover and show that—even when a threshold value for crown cover percent is given and crown cover is perfectly mapped—forest cover figures vary considerably as a function of the size of the reference area, which in turn interacts with the degree of fragmentation of the studied landscape. Crown cover percent is in general a meaningful criterion for the definition of forest land as it is directly related to various forest variables, e.g., productivity, microclimate, and carbon storage. But it is an obvious conclusion from this study that if minimum crown cover is among the criteria of a forest definition, it needs to be complemented by a clear definition of how crown cover percent is measured. That refers in particular to the definition of the reference area.

Spatial resolution plays, according to the findings of this study, only a minor role. For crown cover thresholds <1 differences in forest cover due to changes of the spatial resolution, simulated by spatial aggregation of pixels, were <10 %, an order of magnitude also reported by Nelson et al. (2009) for majority-based aggregation of Landsat images. The minor resolution effect can be highly relevant for remote sensing-based forest monitoring where information from sensors with different resolutions is combined. Such situations are currently prominent in the REDD context where time series are compiled by combining historic lower resolution data (e.g., Landsat) with current high-resolution data. Even though aggregating binary maps is not fully comparable to aggregating spectral information, as it would be the case for real images, the simulation gave an indication that uncertainties caused by the forest definition, including the corresponding reference areas, can exceed those from changes in the spatial resolution even though both can affect the forest cover estimates.

Another major issue that requires closer investigation is the compatibility of forest land definitions when assessed in remotely sensed imagery versus field inventories. This question, although omnipresent, is only rarely addressed explicitly (e.g., Blackard et al. 2008; Wulder et al. 2008). The two data sources are so different with respect to all major characteristics that ensuring compatibility of definitions is a challenge and hard to verify. Two points are noteworthy: First, the definition of the reference area will determine the area that needs to be observed to make the forest/non-forest decision. Using image processing techniques, almost any reference area can be implemented. For terrestrial surveys, reference areas corresponding to commonly used field plot designs are preferable. Second, the definition of forest edge needs to be compatible for both data sources. Implementation of forest edge definitions in forest mapping is an issue which appears not to have been discussed much in the context of forest definitions. In this study, we presented a forest edge definition which combines the concept of minimum crown cover percent with a fixed reference area. This resulted in an operational forest edge definition which could easily be implemented in remotely sensed data using moving window techniques. However, applying this concept in a terrestrial survey is challenging as the point where crown cover percent falls below the minimum threshold can hardly be determined in field. Other forest edge concepts are in use (e.g., Zingg and Bachofen 1988; Traub et al. 2000) but it appears that the measurement rules where to exactly draw the forest boundary line are largely neglected. Kleinn et al. (2011) research such measurement rules for forest boundary and their effects on forest edge length estimation. A novel approach was presented by Eysn et al. (2012) who defined the reference area for crown cover measurements using a triangulation concept. In this approach, no size of the reference area has to be selected as it is determined by the position of the trees. However, the implementation of the triangulation concept in terrestrial surveys seems to be challenging and less compatible with existing forest inventory schemes.

Considering the overall goal of the UN-FCCC and related programs, to reduce atmospheric concentrations of greenhouse gases, a land cover classification is not mandatory for monitoring carbon stocks and their dynamics. It must rather be seen as an instrument: from a scientific point of view, monitoring of the carbon dynamics could be done in a consistent manner over all lands without an a priori breakdown into artificial classes (such as “forest land,” “grassland,” etc.). From a REDD implementation point of view, however, land cover classes are probably an essential instrument. MRV is most easily done and best understood when referring to classes which define geographical units at the same time. For international forest policy that aims to implement trading schemes, a further harmonization of the definition of these classes is required.

It is unlikely that all nations will agree upon one unique global forest definition, as manifold forest types exist which can hardly be covered by one definition. More likely, a range of values for specific criteria will be accepted as it is already the case under the clean development mechanism scheme where crown cover thresholds between 10 and 30 % are accepted (UNFCC 2002). Then, a model that predicts forest area as a function of the crown cover percent and the corresponding reference area is needed for harmonization of forest area statistics that were compiled from different threshold values and reference areas. The COST E43 initiative planned to establish such models, referred to as *bridges* (Vidal et al. 2008), on the European level. Our results indicate that building such a *bridge* as a linear model is critical as the effects are nonlinear, although a high coefficient of determination was observed for compact landscapes. Further, we found that more factors than the studied landscape properties (e.g., fragmentation, crown proportion) influence the relationship between crown cover, reference area, and forest cover. Landscape metrics, which describe the landscape spatial pattern, could be a starting point for further research.

It is fully acknowledged that the forest definition issue is much more complex than only using the minimum crown cover criterion as in this study which is in agreement to Putz and Redford (2010) and Lund (2002). Various other criteria need to be applied simultaneously, as pointed out in the introductory sections; any conflicts emerging from contradictions of these criteria need then to be clarified in order to achieve an unambiguous definition. At the end, the question remains whether natural sciences can provide a forest definition which is perfectly unambiguous, independently of the data source used and which is applicable also in practice. Using crown cover percent as a criterion to define forest land is a pragmatic approach with uncertainties especially when threshold values significantly differ from 50 %, a common situation in internationally used forest definitions. Defining a reference area for crown cover percent measurement could reduce these uncertainties even though some vagueness remains.
